# Identifying the Drivers Related to Animal Reservoirs, Environment, and Socio-Demography of Human Leptospirosis in Different Community Types of Southern Chile: An Application of Machine Learning Algorithm in One Health Perspective

**DOI:** 10.3390/pathogens13080687

**Published:** 2024-08-14

**Authors:** Himel Talukder, Claudia Muñoz-Zanzi, Miguel Salgado, Sergey Berg, Anni Yang

**Affiliations:** 1 Department of Geography and Environmental Sustainability, University of Oklahoma, Norman, OK 73019, USA; himel.talukder-1@ou.edu; 2 Division of Environmental Health Sciences, School of Public Health, University of Minnesota, Minneapolis, MN 55454, USA; 3 Preventive Veterinary Medicine Department, Faculty of Veterinary Sciences, Universidad Austral de Chile, Valdivia 5090000, Chile; miguelsalgado@uach.cl; 4 Department of Computer & Information Science, University of St. Thomas, St. Paul, MN 55105, USA; berg@stthomas.edu

**Keywords:** leptospirosis, communities, environmental drivers, animal reservoirs, One Health, extreme gradient boosting, eco-epidemiology

## Abstract

Leptospirosis is a zoonosis with global public health impact, particularly in poor socio-economic settings in tropical regions. Transmitted through urine-contaminated water or soil from rodents, dogs, and livestock, leptospirosis causes over a million clinical cases annually. Risk factors include outdoor activities, livestock production, and substandard housing that foster high densities of animal reservoirs. This One Health study in southern Chile examined *Leptospira* serological evidence of exposure in people from urban slums, semi-rural settings, and farm settings, using the Extreme Gradient Boosting algorithm to identify key influencing factors. In urban slums, age, shrub terrain, distance to *Leptospira*-positive households, and neighborhood housing density were contributing factors. Human exposure in semi-rural communities was linked to environmental factors (trees, shrubs, and lower vegetation terrain) and animal variables (*Leptospira*-positive dogs and rodents and proximity to *Leptospira*-positive households). On farms, dog counts, animal *Leptospira* prevalence, and proximity to *Leptospira*-contaminated water samples were significant drivers. The study underscores that disease dynamics vary across landscapes, with distinct drivers in each community setting. This case study demonstrates how the integration of machine learning with comprehensive cross-sectional epidemiological and geospatial data provides valuable insights into leptospirosis eco-epidemiology. These insights are crucial for informing targeted public health strategies and generating hypotheses for future research.

## 1. Introduction

Leptospirosis, a zoonotic disease of global distribution caused by the pathogenic bacterial species *Leptospira*, poses a significant health risk to both humans and animals [1]. In humans, leptospirosis can cause asymptomatic infection, flu-like illness, or sometimes jaundice, kidney failure, meningitis, or even death [2]. Globally, it has been estimated that leptospirosis causes around a million clinical cases and 58,000 deaths each year, but, due to poor diagnosis and reporting, the actual burden is unknown [3]. The transmission of *Leptospira* is a multifaceted process that includes a diverse range of hosts and reservoirs and operates through both direct and indirect pathways. Humans may acquire infections either through direct contact with infected animals, both wildlife and domestic animals, or indirectly through exposure to contaminated environments (soil, water) [4]. It is considered an occupational hazard related to activities such as agriculture, sewage management, and animal husbandry [5], as well as an infection associated with routine domestic or recreational activities that put individuals in contact with a *Leptospira*-contaminated environment. Rodents (rats, mice) serve as primary synanthropic reservoirs, although other wildlife species such as raccoons, skunks, opossums, foxes, and deer can also become infected [6]. Dogs transmit the pathogen to humans through either direct contact, urine-infected materials, or the environment [7]. Domesticated farm animals like cattle and sheep can also act as carriers of *Leptospira*, and people can become infected by direct contact with blood, aborted fetuses, vaginal discharge, or calving products from infected animals, as well as indirectly through the urine-contaminated farm environment [8]. Human-to-human infection is also possible via breast milk or sexual contact but is very rare [9,10]. More importantly, people can become infected when exposed to water or soil that is contaminated with *Leptospira* [11]. Communities with low socio-economic conditions are vulnerable to exposure given the inadequate sanitation and lack of safe drinking water [12]. Tropical climate areas where there is frequent flooding caused by high rainfall and natural disasters often result in a high risk of *Leptospira* infection [13,14]. Moreover, humidity and warm temperatures in tropical areas cause the pathogen to persist longer in the environment, specifically in soil, which in turn increases the likelihood of exposure [15]. Biofilms play a significant role in the persistence of *Leptospira* in the environment. Biofilms provide a protective niche that enhances the survival of *Leptospira* in aquatic and soil environments, making it more resilient to environmental stressors and increasing the risk of transmission [13,15]. The multifaceted nature of leptospirosis is driven by a variety of factors, encompassing ecological, animal, and anthropogenic elements. Geographical differences further contribute to the dynamic nature of the transmission, indicating that the intricate web of factors influencing the disease may differ significantly from one community to another. Understanding the interactions among these elements is crucial for developing targeted and effective strategies to mitigate the impact of leptospirosis in different regions. However, the existing literature is scarce about how leptospirosis can be present in different community settings and the unique relationships between the various transmission drivers.

The growing body of research on leptospirosis prevalence highlights the lack of a comprehensive investigation framework. While numerous studies have explored the prevalence of leptospirosis within different populations, a noticeable limitation arises from the absence of simultaneous consideration of human, animal, and environmental factors. The One Health approach advocates for an integrated and interdisciplinary perspective that acknowledges the interconnectedness of human, animal, and environmental health. Utilizing the principles of One Health in leptospirosis research facilitates a more holistic understanding of disease dynamics and transmission pathways. By scrutinizing the complex interactions among humans, animals, and their shared environment through the adoption of a One Health framework, researchers can gain insight into the intricate epidemiology of leptospirosis and devise more efficacious prevention and control measures.

The application of machine learning in epidemiological studies has grown significantly, offering robust tools for analyzing large and complex datasets [16,17]. However, its use within the One Health framework remains limited [18,19]. Given the diversity of pathogens and hosts in One Health diseases, the complexities of their transmission cycles, and the multiple drivers of emergence, no single data source or technique is sufficient for detecting and understanding those emerging infectious diseases. Traditional statistical models often fail to capture the complex relationships among multiple streams of diverse information [18]. Thus, it is crucial to promote and highlight the use of advanced computational frameworks such as machine learning and artificial intelligence to harmonize and analyze large, dynamic, and heterogeneous data streams in One Health research [20,21].

Here, we employed an existing dataset to showcase how machine learning algorithms can be implemented within a One Health framework. The data presented herein constitute an ideal representation of such a framework by integrating leptospirosis data from rodents, dogs, and the environment (all previously published), along with new livestock and human seroprevalence data from an eco-epidemiological study conducted in Chile [11,22,23]. The Los Rios region in south-central Chile, a predominantly agricultural and farming area juxtaposed with scattered urban settlements, has been the location of several leptospirosis studies [24]. A previous survey targeting individuals with occupational hazards revealed a seroprevalence of around 22%, underscoring the need to elucidate disease dynamics and effective control measures [25]. Additionally, a survey conducted on dogs in the area reported a leptospirosis seroprevalence of 25%, with variations across different community types, highlighting the interconnectedness of human-influenced environments and animal health [26]. Rodents in the region were shown to have kidney carriage of around 20%, further emphasizing the multifaceted nature of leptospirosis transmission pathways in the Chilean site [27].

Using a One Health framework, the objective of this study was to identify the intricate relationships between human *Leptospira* exposure and different drivers, including household characteristics, animal reservoirs, environmental conditions, and water sources, in three distinct community settings in the Los Rios region of Chile: urban slums, semi-rural communities, and farm communities. We hypothesized that the drivers of human *Leptospira* exposure would be different in those three communities, given the different environmental settings and compositions of animal reservoirs. Specifically, we expected that rodent-related variables and household conditions would drive human *Leptospira* exposure in urban slum communities, while exposure in semi-rural areas would be impacted by environmental conditions and household variables. In farm communities, we expected that livestock and wildlife-related variables would play an important role in driving human *Leptospira* exposure.

## 2. Materials and Methods

### 2.1. Study Area

**Figure 1:**
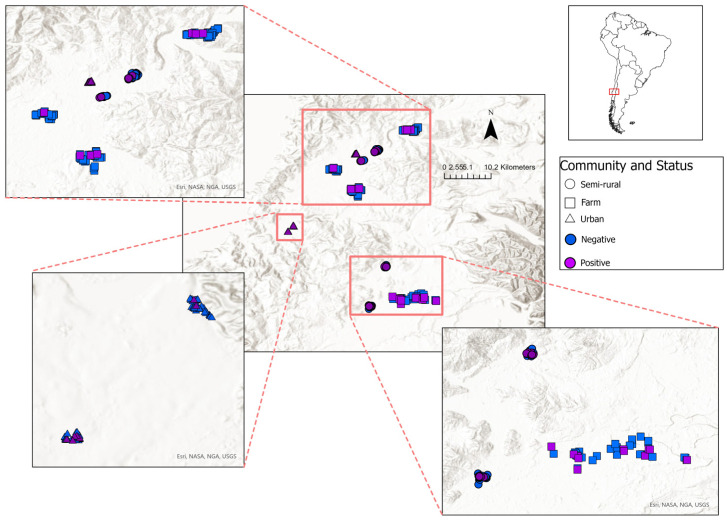
Study area and distribution of leptospirosis seropositive (purple) and seronegative (blue) participants across the various community types indicated by shape (semi-rural: circle, farm: square, urban slums: triangle).

The study design and data come from a study on the eco-epidemiology of leptospirosis conducted in the Los Rios region in south-central Chile (latitude: 39°15′ S–40°33′ S, longitude: 73°43′ W–71°35′ W) [11,22]. The climate of the region is characterized as temperate rainforest, with an annual cumulative rainfall of 2588 mm and a range of 1200 mm in the central valley up to 5000 mm in the Andes Mountains. The average temperature has less variation throughout the year, with 17 °C in summer and 8 °C in winter [22,27]. Communities were selected to represent three community types: (i) urban slums: informal settlements in the outskirts of a major city characterized by substandard housing; (ii) semi-rural: rural community settlements away from major cities where households are clustered together; and (iii) farm: dispersed households, typically small family farms, located in a specific rural locality. Communities were chosen from areas that have the highest number of settlements in the region, specifically the central valley and the vicinity of the region’s capital (see Figure 1). Most of the communities were located within an elevation of 0–100 m above sea level, except for two households in farm communities, which were at 100–200 m above sea level [3,27]. Individual households within each community were selected randomly and enrolled based on their willingness to participate in all components of the study, which included rodent trapping, sampling of domestic animals, sampling of surface water sources in the peri-domestic environment, and sampling of household members. Written informed consent was obtained from each household member who provided a blood specimen for serologic analysis and from the head of household who authorized rodent trapping and the collection of blood samples from their domestic animals.

### 2.2. Data Collection

This research is a case study utilizing the One Health framework to investigate the drivers of human *Leptospira* exposure in diverse community settings. The data collection for the eco-epidemiology study was conducted between August 2010 and March 2012 and involved 422 households from 12 communities, 4 of each community type. A questionnaire survey was carried out to obtain information on socio-demographic characteristics, housing conditions, the presence of animals, and to characterize each sample.

**Leptospirosis status in people and domestic animals**: Data available included the *Leptospira* exposure status of 907 people. Household members 13 years old and older, apparently healthy, who consented to participate provided a one-time serum sample to measure *Leptospira*-specific antibodies. Dogs and livestock present at the residence were also sampled to obtain serum for *Leptospira* antibody testing.

The microscopic agglutination test (MAT) with a panel of 20 serovars, representing 17 serogroups, was run on serum samples at Austral University, Chile *(L. interrogans* serovars Australis, Bratislava, Autumnalis, Bataviae, Canicola, Djasiman, Grippotyphosa, Icterohaemorrhagiae, Mankarso, Pomona, Pyrogenes, and Wolffi, *L. borgpetersenii* serovars Ballum, Javanica, and Tarassovi, *L. kirschneri* serovar Cynopteri, *L. santarosai* serovars Borincana, Alexi, and Georgia, and *L. weilii* serovar Celledoni) as described previously [26]. Titers of 1:100 or higher were considered positive to classify each individual (people and animals) as seropositive or seronegative for *Leptospira*. We calculated the cumulative number of seropositive animals for cattle (*bov_com_pos*), sheep (*ovi_com_pos*), dogs (*dog_com_pos*), and all animals (*animal_pos_com*) in each community.

**Leptospirosis status in rodents**: Trapped rodents were euthanized, and kidneys were tested for the presence of *Leptospira* DNA using Polymerase Chain Reaction (PCR) as previously described [22]. Test results were used to create a variable representing the number of households within 100 m with positive rodents weighted inversely by the distance from the house (*distance_pos_rod*).

***Leptospira* contamination in the peridomestic environment**: Surface water samples were collected from various sources and locations in the household environment and tested for the presence of pathogenic *Leptospira* DNA using PCR as previously described [22]. Results were then used to calculate variables that represent the proportion of positive puddle samples in the community (*puddle_pos*), the proportion of positive samples considering all water source types (puddles, rivers, wells, ponds, etc.) in the community (*water_prev_com*), and the number of households within 100 m with positive water samples weighted inversely by distance from the house (*distance_pos_water*).

**Participant characteristics**: A questionnaire was used to collect individual socio-demographic characteristics (*age*, *sex*) and behaviors that could expose people to *Leptospira,* such as gardening (*garden*), swimming (*swim*), cleaning animal barns (*clean_barn*), cleaning sewers (*clean_drain*), water drainage (*clean_water_drain*), slaughtering animals (*slaughter*), milking (*milking*), and/or cleaning after animal birth (*clean_birth*).

**Household animal characteristics**: Rodent presence was obtained based on trapping efforts as the number of rodents trapped at the household (*rodent_count*) and the total for each community (*rodent_count_comm*), as previously described [22]. A questionnaire was used to collect data on the reported presence of rodents (e.g., observing rodents, seeing rodent droppings, seeing or smelling rodent urine, whether gnawed boxes, food, or wood were found, holes in the walls were found, or hearing rodent noises) [22], as well as domestic animal-related variables such as the total number of cattle, sheep, and dogs in the household.

**Spatial and environmental variables**: Geospatial variables from multiple sources were used to describe the environmental settings of each community. Details on how variables were generated have been described previously [22,27]. Variables used for this analysis included the number of houses within a 100-m radius (*house*) and the number of buildings within a 100-m radius (*buildings*). Land cover was defined as the dominated terrain within the surrounding radius and categorized as tree canopy (*tree*), lower vegetation (*lowveg*), shrub (*shrub*), or barren space (*field*). Bioclimatic variables used in this study to represent the climatic conditions of the study area (*bio1*, *bio2*, *bio12*, *bio15*) were collected from WorldClim (worldclim.org, accessed on 23 October 2023) [28].

All human participants provided written consent to participate in this study, including the disclosure of their domestic and livestock information. The study protocol was approved by the University of Minnesota’s Institutional Review Board (No. 0903 M62042), the Institutional Animal Care and Use Committee (No. 0904A63201), and the Austral University’s Human and Animal Ethics Committee (No. 01/09). More details about data collection can be found in [11,22,26,27]. See Table 1 for further information on data descriptions and sources.

### 2.3. Extreme Gradient Boosting Model

XGBoost is a high-performing gradient classification and regression boosting machine learning algorithm that is widely used in epidemiology and disease ecology for tasks such as predicting disease outbreaks, identifying risk factors, and modeling pathogen transmission dynamics, demonstrating its effectiveness in handling complex biological and environmental data [29,30,31]. XGBoost is an ensemble machine learning algorithm that combines multiple weak learners, typically decision trees, to form a strong predictive model [29,32]. The algorithm works by sequentially adding trees to minimize the errors of the existing ensemble through gradient boosting. In each iteration, a new tree is trained to correct the residuals (errors) of the previous trees, which helps in capturing complex patterns in the data [29]. XGBoost employs regularization techniques such as L1 (Lasso) and L2 (Ridge) to prevent overfitting, ensuring the model remains generalizable [31,32]. The algorithm also uses advanced optimization methods like parallel tree construction and efficient handling of missing data to enhance computational efficiency [30,31]. These features make XGBoost particularly effective for handling large datasets with complex relationships between features [31]. This method is well suited for our analysis because it identifies complex and non-linear relationships among the drivers and *Leptospira* exposure and adjusts the collinearity between the drivers [33].

We run separate XGBoost models for the three different community types. For each model fitting, we randomly split the observations into training and testing sets under the ratio of 80% and 20%. The training dataset was used to run the models and identify the drivers, while the testing dataset was used to estimate the accuracy of the model. Additionally, we utilized cross-validation to ensure that our model was not overfitting and to validate the model’s performance across different subsets of data. This involved partitioning the training data into multiple folds and training multiple models to evaluate their consistency.

The performance of XGBoost can be sensitive to its hyperparameters [34]. After splitting, we tuned a variety of hyperparameters to optimize the performance of the model. A matrix of hyperparameters was provided by using the *expand.grid* function to find the best combination of the hyperparameters [35]. Specifically, the maximum tree depth, learning rate, and gamma were adjusted here, given that these variables generally exhibit the most significant impact on model performance [36]. Furthermore, we accounted for unbalanced classes, which refers to an imbalance between the number of positive and negative cases, by adjusting the model parameter ‘*scale_pos_weight*’ [37]. This is estimated as the total number of negative cases divided by the total number of positive cases (see Table 2 for detailed information about the parameterization). The final XGBoost classification model was fitted using those tuned hyperparameters. Early stopping was also implemented to prevent overfitting by halting training once the model’s performance on the validation set stopped improving. To account for stochasticity in the random split of training and testing sets in model development, we performed 2000 iterations of our data splits and performed the final splitting processes for them. This allowed us to use an ensemble modeling approach that incorporated information and uncertainties from multiple random split scenarios and created confidence intervals for evaluating the model and determining the importance of the drivers.

We also generated the variable importance (‘gain score’ represents how much a variable contributes to enhancing the model’s predictions) for every model iteration and took the average to show the most important drivers of leptospirosis in three community types and the partial dependency plots to show the relationship between potential drivers and *Leptospira* exposure. To evaluate the performance of the final model, we used the area under the receiver operating characteristics curve (AUC) value, which assesses how well the model can distinguish positive and negative cases [38]. The XGBoost model development and the associated analyses were performed using the “*XGBoost*” package in R version 4.3.1 [39,40].

## 3. Results

### 3.1. Seroprevalence of Leptospirosis

**Figure 2:**
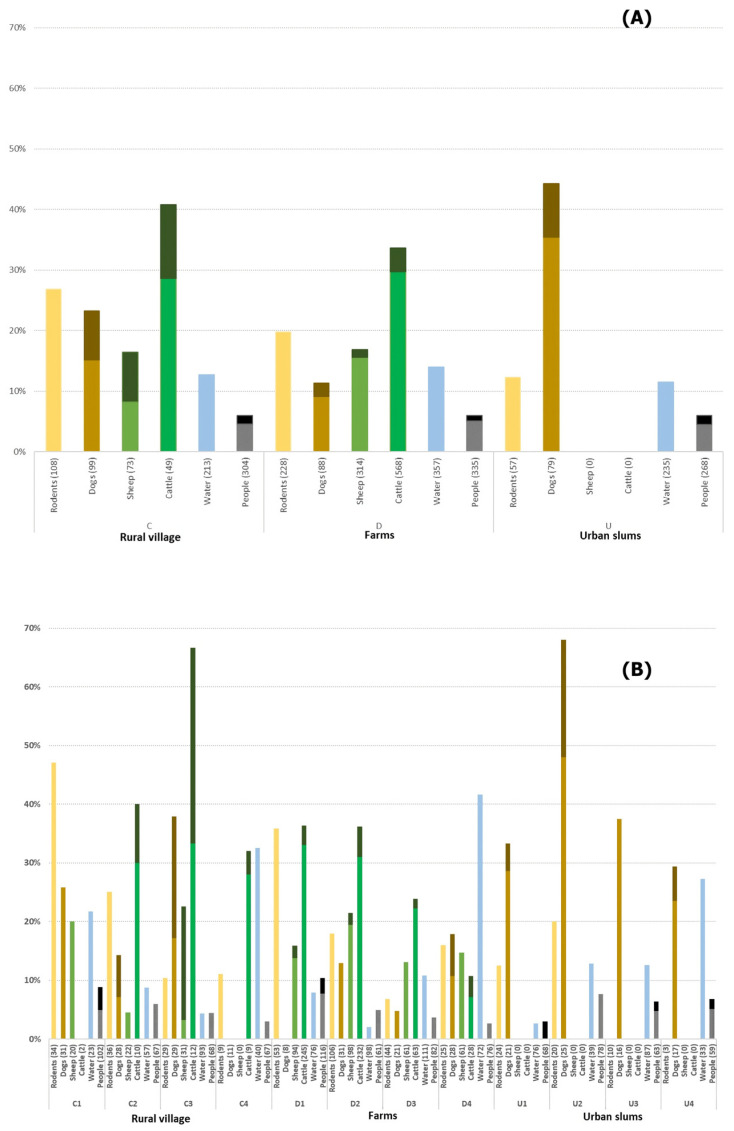
*Leptospira* positivity distribution in rodents (PCR kidney carriage positivity), domestic animals (MAT seropositivity), water (PCR positivity), and people among MAT seropositivity (**A**) by community type and (**B**) in each of the 12 communities, three within each community type. Darker color in a bar represents the proportion of individuals with a MAT titer ≥ 1:400.

Evidence of *Leptospira* exposure was evident in all community types, with an overall seroprevalence in people of 6.0%. Seroprevalence was also 6.0% for each community type, while ranging by community from 3.7% to 10.3%. *Leptospira* occurrence was high, with 20.4% of rodent kidney carriers; 5.6% in urban slums; 19.7% in semi-rural areas; and 25.7% in farm communities. Overall, 13.5% of water samples were classified as PCR-positive. Among dogs owned by household members, 26.8% were seropositive, with a marked increase in urban dogs (45.1%) compared with rural dogs (22.3%). Sheep and cattle were the main livestock in the area, with overall seroprevalences of 16.5% and 31.2%, respectively (Figure 2).

Overall, our study revealed varying seroprevalence in people across different demographic and behavioral categories. Males exhibited a higher seroprevalence (6.5%) than females (5.6%), while individuals engaging in swimming activities showed a higher seroprevalence (6.8%) compared with non-swimmers (4.0%). People living in households harboring positive rodents and dogs had greater seroprevalences (7.1% and 7.8%, respectively) than those without such animals (5.0% and 5.6%, respectively). Additionally, gardening and barn cleaning activities were associated with increased seroprevalence (6.7% and 6.8%, respectively) compared with individuals who did not perform these tasks (4.1% and 5.4%, respectively, missing % for those). People who reported activities involving slaughtering and milking animals displayed higher seroprevalence (7.3% and 8.3%, respectively) than those who did not (5.7% and 5.8%, respectively) (Table A1). Notably, the lowest seroprevalence was observed among participants involved in sewage drain cleaning activities (2.3%).

### 3.2. Urban Slum Community

**Figure 3:**
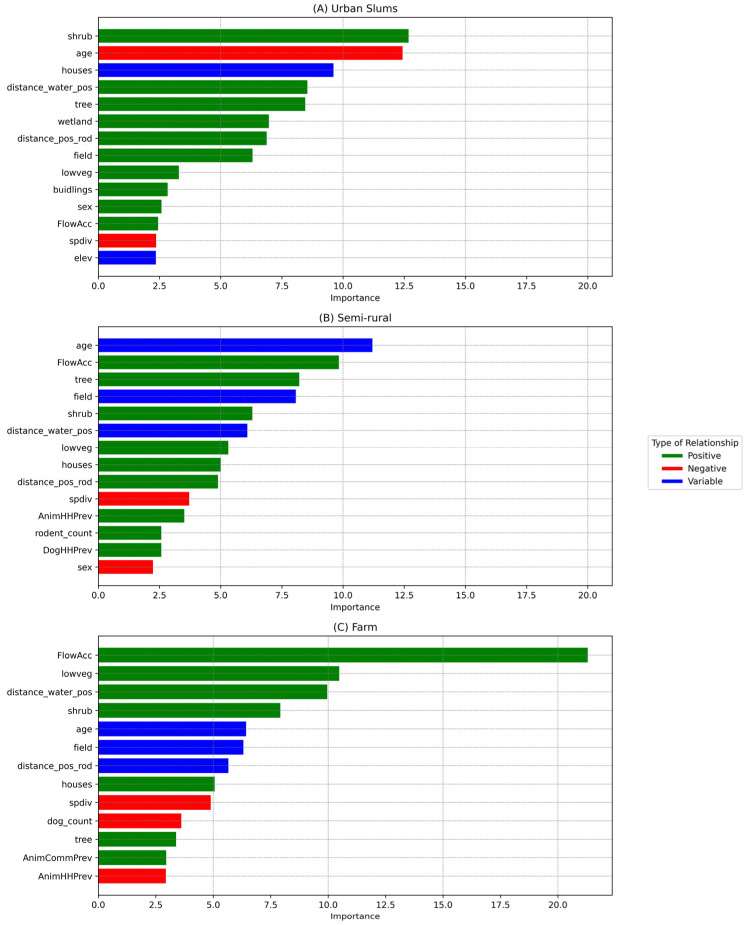
Variable importance plot for the model results of the three community types: (**A**) urban slums, (**B**) semi-rural, and (**C**) farm. Variables with more than a 2% importance frequency score have been shown. The bars were color-coded for ease of interpretation of the overall trend in the predicted relationship between each variable and human exposure probability across the three different community types. Note that the relationship types depicted here were derived visually from Figure 4, Figure 5 and Figure 6 and were simplified to overall positive or negative, but actual relationships are non-linear.

The final XGBoost model for urban slum communities that was used to predict the seropositive and seronegative participants for the testing data performs well with an average AUC value of 95.09% (range: 87.08–98.36%). The most important drivers in urban slum communities were the square meters of shrub terrain in a 100-m radius (*shrub*), followed by age, and the number of houses in a 100-m radius (*houses*) (Figure 3A).

**Figure 4:**
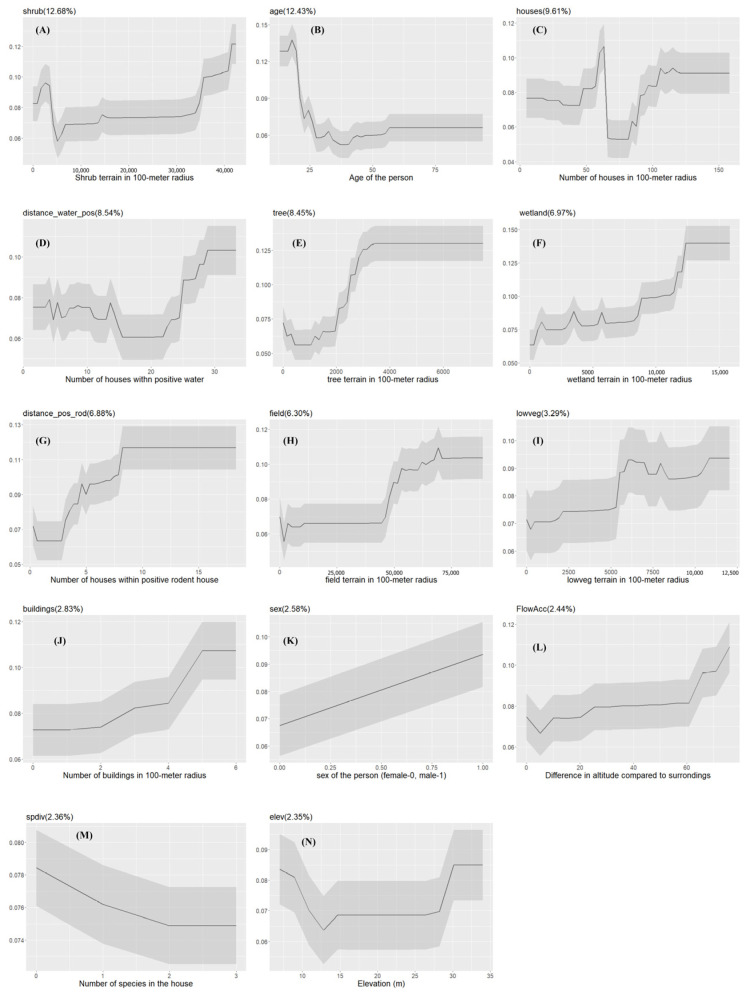
The partial dependency plots of covariates used in the model for urban slum communities. The gray shading area indicates the confidence intervals derived from the model iterations. The variable importance score is reported in the parentheses ((**A**–**N**): partial dependency of each variable in the model).

The probability of *Leptospira* exposure, in general, increases as the areas of shrub terrain within a 100-m radius increase (Figure 4). Males have a higher probability of getting exposed to *Leptospira* than females.

The probability of exposure is positively correlated with the number of houses in a 100-m radius. Additionally, model results revealed that environmental drivers such as areas of wetlands (*wetland*), tree terrain (*tree*), and low vegetation (*lowveg*) in a 100-m radius can positively impact *Leptospira* exposure. The local ecology of *Leptospira* was reflected in the relationship between the probability of human exposure and the number of households within 100 m with positive water samples (*distance_water_pos*) and the number of households within 100 m with positive rodents (*distance_pos_rod*) (Figure 4).

### 3.3. Semi-Rural Community

**Figure 5:**
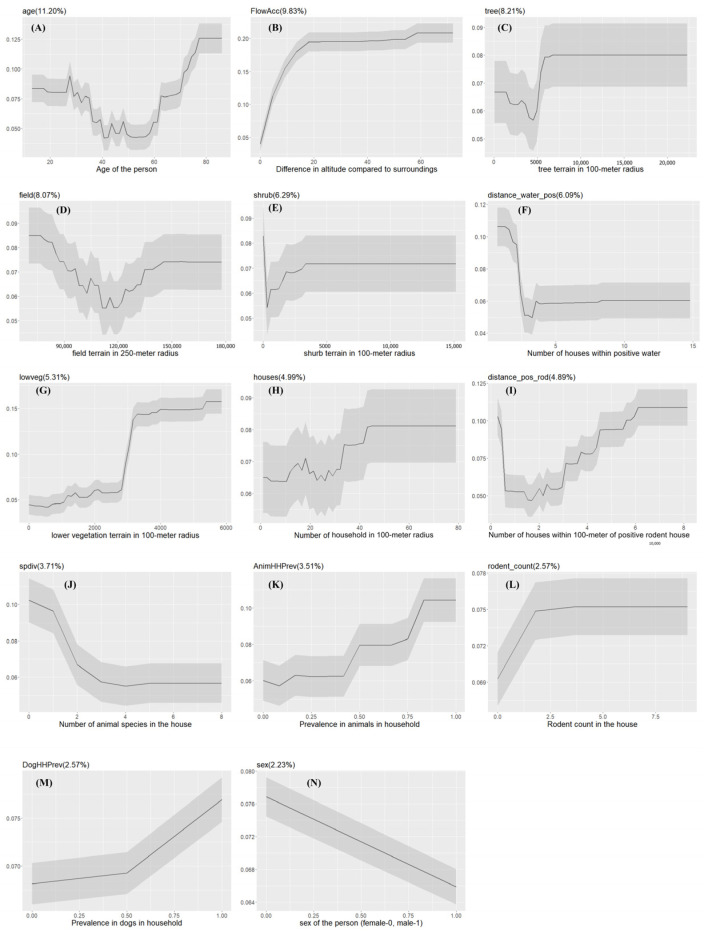
The partial dependency plots of covariates used in the model for semi-rural communities. The gray shading area indicates the confidence intervals derived from the model iterations. The variable importance score is reported in the parentheses ((**A**–**N**): partial dependency of each variable in the model).

The AUC value from model performance for the model of semi-rural communities was 88.27% (range: 82.63–90.04%). The most important drivers of *Leptospira* exposure in semi-rural communities were age, slope of the terrain (*FlowAcc*), and *tree* and *field* areas (Figure 3B). The probability of *Leptospira* exposure was higher among the youngest and the oldest people, in particular those older than 60 years old (Figure 5). The probability of exposure increased when the slope of the terrain increased. There was a higher exposure probability when there was high tree coverage, while this effect was opposite for field area coverage.

With a relatively low importance score, several animal-related drivers emerged in the model (Figure 3B). Household seroprevalence of *Leptospira* in animals (*AnimHHPrev*), trapped rodent count (*rodent_count*), and household seroprevalence of *Leptospira* in dogs (*DogHHPrev*) were positively associated with exposure probability, while species diversity (*spdiv*) was negatively associated. Exposure probability generally increased as the number of households within 100 m with positive rodents (*distance_pos_rod*) increased (Figure 5).

### 3.4. Farm Community

**Figure 6:**
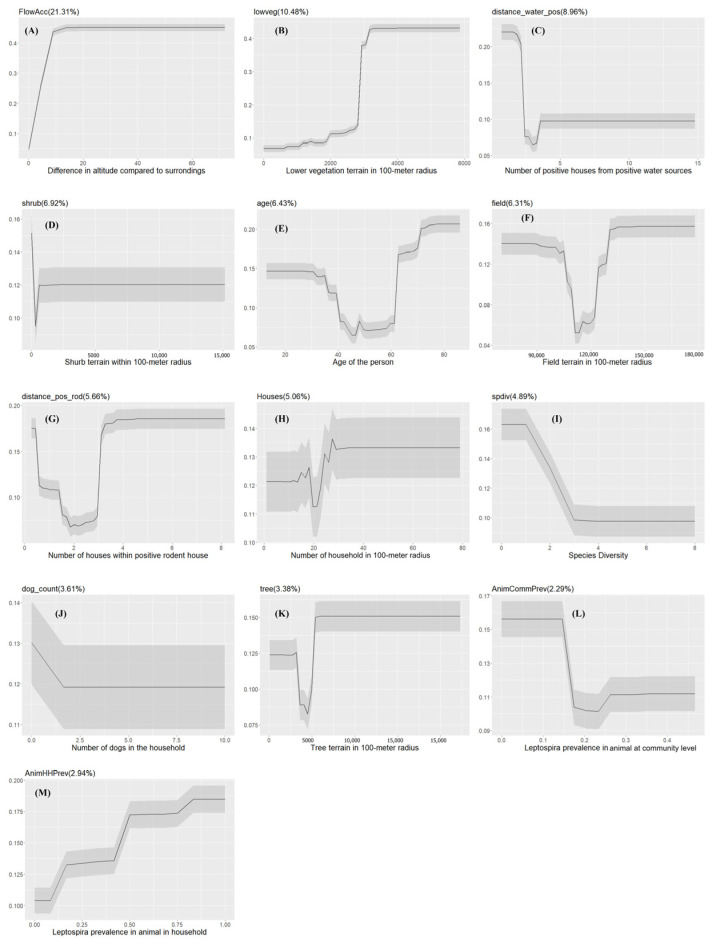
The partial dependency plots of covariates used in the model for farm communities. The gray shading area indicates the confidence intervals derived from the model iterations. The variable importance score is reported in the parentheses ((**A**–**M**): partial dependency of each variable in the model).

The XGBoost model showed high prediction of seropositive and seronegative individuals with an AUC value of 91.74% (range: 84.56–95.13%). Slope of the terrain (*FlowAcc*), low vegetation area (*lowveg*), and the number of households within 100 m with positive water samples (*distance_water_pos*) were significantly affecting human *Leptospira* exposure (Figure 3C). The probability of *Leptospira* exposure was positively correlated with terrain slope and low vegetation and negatively correlated with the number of households within 100 m with positive water samples (Figure 6).

Other results showed that the exposure probability increased with the increasing seroprevalence of *Leptospira* among animals in a household (*AnimHHPrev*), while species diversity (*spdiv*), the number of dogs in the household (*dog_count*), and community-level seroprevalence in animals (*AnimCommPrev*) were negatively associated with the probability of *Leptospira* exposure. Age had a U-shape type of relationship, similar to the semi-rural community type, indicating a higher exposure probability for the younger and older ages. For the number of households within 100 m with positive rodents (*distance_pos_rod*), the relationship was variable, but it increased sharply when there were three or more households within 100 m with positive rodents (Figure 6).

## 4. Discussion

Leptospirosis, a zoonotic disease caused by the pathogenic spirochetes of the genus *Leptospira*, poses significant public health challenges worldwide, especially in tropical regions [41,42]. It is also present in temperate regions, and, using One Health principles, our case study unveiled the intricate landscape of leptospirosis transmission across varied community settings in Chile. We found distinct seroprevalences and risk factors associated with demographic, behavioral, environmental, and animal-related variables in those communities. Urban slum areas showed higher exposure probabilities linked to environmental factors like shrub terrain and positive water samples, while semi-rural and farm communities exhibited different patterns influenced by age, household characteristics, and animal prevalence. These findings shed light on the multifaceted nature of leptospirosis transmission, informing targeted interventions for reducing human exposure and enhancing public health efforts.

Our research combined data from systematic efforts to detect leptospirosis in humans, animals, and the peri-domestic environment, unveiling the presence of *Leptospira* across the distinct community types in the study area of Chile and with varying seroprevalences among humans and domestic animals, rodent kidney carriages, and environmental contamination. The overall 6% seroprevalence in humans is similar to several studies in Colombia and Mexico in similar socio-economic settings [43,44,45]. Previous studies found that features of inadequate living conditions such as dirt floors, proximity to sewage, and absence of proper sanitation, as well as behavioral factors such as walking barefoot, having uncovered wounds, and gathering firewood, are contributors to exposure [15]; however, our modeling did not reveal any individual behaviors as significant risk factors. This may be because disease transmission is primarily determined by the living environment, where external factors can have a more significant impact on disease spread, overshadowing the influence of individual behaviors. It could also be because MAT antibodies are evidence of an exposure in the past that may not be reflected in the behaviors reported in the survey. Based on our overall descriptive analysis, males exhibited a higher seroprevalence (6.5%) compared with females (5.6%), suggesting potential gender-specific differences in exposure or susceptibility to infection, which have often been reported in the existing literature [46,47,48]. However, when analyzing by community type, our XGBoost models revealed contrasting effects, in which men had a higher exposure probability than women in urban slum communities, but women had a higher exposure probability than men in semi-rural communities. This finding may reflect differences in behavioral patterns, occupational activities, or exposure to contaminated environments between genders [49]. Models consistently revealed age as an important factor, but with different relationships. There was a negative association with age in urban slum communities, with a higher probability at younger ages, but it was U-shaped in semi-rural and rural communities. The different trends could reflect different demographics and associated activities in the communities. For example, study participants from urban slum communities tended to be younger [50], and the higher probability among this group could reflect their greater representation. In semi-rural and rural areas, a higher exposure probability among the youngest (<40 years) and the oldest (>60 years) ages could reflect common recreational, occupational, and domestic activities with exposure to a contaminated environment or infected farm animals, such as swimming, gardening, or livestock management. The high exposure probability among older adults may also reflect a longer period at risk of becoming exposed.

Several environmental variables, such as shrub terrain, wetlands, tree terrain, lower vegetation, and field terrain, showed an impact on the likelihood of *Leptospira* exposure, suggesting the importance of considering landscape heterogeneity when assessing exposure risk across different types of communities. The identified positive correlation with vegetation covers surrounding households, such as the presence of trees and lower vegetation (i.e., bushes) in all community types and shrubs in urban communities, highlights the interconnectedness of wildlife habitats and leptospirosis risk [51]. Those land covers can act as critical habitats for wildlife reservoirs, notably rodents, increasing the likelihood of human–animal contact and subsequent disease transmission [52,53,54]. Additionally, the shaded, humid conditions under vegetation cover can modify microclimatic conditions by trapping and retaining moisture in the soil, creating damp conditions that are ideal for the survival of *Leptospira* outside of its hosts. This extended survival in moist soil increases the duration during which humans can encounter *Leptospira*, further facilitating transmission [51,55,56]. Furthermore, the presence of wetlands was positively associated with the likelihood of exposure in semi-rural and farm communities, which is consistent with the notion that they can act as reservoirs for *Leptospira* [53,55], facilitating its survival and dissemination through water-borne transmission [5,52]. The presence of open fields can also be a contributing factor because it can lead to trash accumulation, attract dogs and rodents, and provide standing water. This factor manifested differently as it was positively correlated with the probability of exposure in urban slums; however, the pattern was different for semi-rural and farm communities, likely due to interactions with other vegetation features and the built-in environment. It is widely recognized that downward slopes enhance surface water flow, leading to the accumulation of stagnant water bodies and moist soil ideal for *Leptospira* survival [48]. The flow accumulation variable used in this analysis was found to be important in the three community types. Derived from slope data, this variable aimed to capture land features that facilitate water movement downhill during rainfall or flooding, which results in the resuspension of *Leptospira* present in the soil and sediment, leading to areas with high environmental contamination and high exposure risk [57].

Leptospirosis has been associated with urbanization, in which the conditions of high-density, low-income housing are highly suitable for *Leptospira* contamination and transmission [58,59,60,61]. This effect of high-density housing was evident across all three community types when measured by the number of houses in a 100-m radius. Additionally, the number of households within a 100-m radius with water samples positive for pathogenic *Leptospira* emerged as a factor positively associated with exposure risk for the urban slum community type, further supporting the idea of exposure risk from a *Leptospira*-contaminated peri-domestic environment in these vulnerable communities [62,63]. However, this relationship differed in semi-rural and farm community types when exposure risk was the highest and there were few houses with positive water samples in the immediate vicinity. This could be an effect of water sources, which are often shared or communal, making contamination more widespread, or that rural households have greater exposure to environmental factors such as open fields or livestock, making localized household contamination less relevant [64]. Additionally, mobility patterns and water usage behaviors in rural communities might lead to a mismatch between household proximity to contaminated water and actual exposure risk [65].

Although animal-related variables were present across all communities, these variables were more evident in semi-rural and farm communities. The variable measuring the number of houses with *Leptospira*-positive rodents in the surrounding area was found to impact exposure risk in all three types of communities. There was a clear positive trend in urban slum communities, which is consistent with the findings regarding water sample contamination. This is also consistent with the general knowledge that synanthropic rodents are ubiquitous reservoirs of *Leptospira* [51]. Although there was a lower importance level in the model results than other variables, dog household seroprevalence in semi-rural communities was associated with increased exposure risk. Dogs are recognized reservoir hosts for leptospirosis, capable of shedding the bacterium in their urine and contaminating the environment [66]. The often limited access to veterinary care and vaccination in low-resource settings leads to underdiagnosis and untreated cases [67,68]. A high seroprevalence of *Leptospira* in dogs within households could be correlated to an increased likelihood of human exposure because of transmission from the dogs or a dog-contaminated environment. Alternatively, both dogs and people could be subject to similar sources of *Leptospira*. Although also of low importance in the modeling results, it is worth noting that the seroprevalence of *Leptospira* in all farm animals within the household was positively associated with exposure risk in semi-rural and farm communities. Households in these communities had a variety of livestock, such as cattle, sheep, and pigs, which can contribute to environmental contamination and pose a risk to household members through occupational and/or domestic activities [69,70,71]. The variable could represent an overall measurement of the underlying *Leptospira* exposure risk at the household level. The inverse relationship observed between increasing species diversity and *Leptospira* exposure, more importantly in rural communities, may reflect several ecological and epidemiological factors. A low diversity of domestic animal species in the farm system investigated here could indicate a higher abundance of more competent hosts that contribute to increased transmission and a higher likelihood of human exposure [54]. This association could also be the result of farm management practices and other activities correlated with both the likelihood of exposure to people and species diversity.

Our study has several limitations. Firstly, the cross-sectional nature of the study design limits our ability to capture the temporal change in the relationship between identified risk factors and *Leptospira* exposure in community participants. Leptospirosis status in the community participants was measured using the presence of antibodies, which indicate an exposure sometime in the past. Although the populations in the study communities were stable regarding time of residence in the sampled locations, some of the factors may not reflect the same conditions as the time of exposure. Additionally, the use of self-reported data and reliance on the recall of the participants may have introduced information bias into reported behaviors. Furthermore, the multi-strain and multi-host transmission dynamics of *Leptospira* are complex, but our study focused solely on the broad relationships between various eco-epidemiological drivers and human exposure. We were not able to investigate the underlying mechanisms or pathways of transmission or how drivers may interact with one another to influence exposure risk. However, the benefit of using cross-sectional sampling, as shown in this case study, is that it allows for a cost-efficient, comprehensive investigation and is particularly useful as an initial approach in areas with limited knowledge of the leptospirosis situation. The findings can then generate hypotheses for future research incorporating longitudinal study designs with incidence cases and genomic approaches for strain identification in humans, animals, and the environment to provide insights into the transmission dynamics of leptospirosis. Lastly, all the data used for this case study were collected during the same time period, which allows for inferences about the observed relationships at that time; however, the findings may not reflect the current situation in the study area.

## 5. Conclusions

Across urban slums, semi-rural, and farm communities, the overall human seroprevalence by community type was similar; however, our study results show the intricate interplay between environmental, socio-demographic, and animal-related factors in shaping leptospirosis transmission dynamics across these different community types. In urban slum areas, densely populated environments and altered landscapes contribute to disease risk, with environmental and rodent-related factors driving the transmission. Similarly, semi-rural communities exhibit complex interactions between environmental features and animal-related variables influencing exposure probability. In contrast, farm communities present unique challenges characterized by the coexistence of agricultural practices and human habitation. Here, environmental factors interact with the presence of livestock to shape disease dynamics. Emphasizing a One Health approach that recognizes the interconnectedness of human, animal, and environmental health is paramount to addressing the complex nature of leptospirosis transmission. The case study presented here is an example of the integration of efforts across disciplines for data collection at the human–environment–animal interface and novel methods such as machine learning for the analysis of complex data. Adopting a holistic approach that considers the health of humans, animals, and ecosystems is essential for achieving sustainable disease control and ensuring the health and prosperity of communities worldwide.

## Figures and Tables

**Table 1:** Description and source of the variables included in the study.

Type	Variable Name	Description	Source
Socio-demographic and household characteristics	*sex*	Sex of the person	Questionnaire
	*age*	Age of the person (in years)	
	*clean_barn*	Person cleans barns	
	*clean_drain*	Person cleans drains in the field	
	*slaughter*	Person butchers meat	
	*milking*	Person milks cows	
	*clean_birth*	Person cleans cow birth products	
	*clean_water_drain*	Person cleans water drains	
	*clean_field*	Person cleans fields	
	*swim*	Person swims	
	*season*	Sampling season	
	*house*	Number of houses within 100-m radius	Derived from worldview-2 satellite imagery
	*buildings*	Number buildings within 100-m radius	
Environmental	*elev*	Altitude of sampled household	Derived from worldview-2 satellite imagery
	*FlowAcc*	Difference in altitude compared with surroundings (higher numbers mean greater slope downward)	
	*tree*	Square meters of tree-dominated terrain within 100-m radius	
	*lowveg*	Square meters of lower-vegetation terrain within 100-m radius (e.g., bushes and other short plants)	
	*shrub*	Square meters of shrub-dominated terrain within 100-m radius	
	*wetland*	Square meters of wetland terrain within 100-m radius	
	*field*	Square meters of field terrain within 250-m radius	
	*bio1*	Annual mean temperature	worldclim.org, accessed on 23 October 2023
	*bio2*	Mean Diurnal Range (mean of monthly (max temp–min temp))	
	*bio12*	Annual Precipitation	
	*bio15*	Precipitation Seasonality (Coefficient of Variation)	
	*puddle_pos_com*	Proportion of *Leptospira*-positive puddles in the community	Laboratory testing
	*water_prev_com*	Proportion of *Leptospira*-positive water samples in the community (all water source types)	
	*distance_pos_water*	Number of households within 100 m with *Leptospira*-positive water samples weighted inversely by distance from house	Derived from worldview-2 satellite imagery
Animal	*rodent_count *	Number of rodents trapped in the household	Questionnaire
	*rod_pos*	Presence of *Leptospira* positive rodents in the household	Derived
	*rodent_count_com*	Number of rodents trapped in the community	Questionnaire
	*RodHHPrev*	*Leptospira* prevalence in rodents at household level	Derived
	*rodent_prev_com*	*Leptospira* prevalence in rodents in the community	Derived
	*distance_pos_rod*	Number of households within 100 m with *Leptospira*-positive rodents weighted inversely by distance from house	Derived from worldview-2 satellite imagery
	*spdiv*	Number of different domestic animal species in the household	Derived
	*bov_count*	Number of bovines in the household	Questionnaire
	*bov_pos*	Presence of seropositive bovines in the household	Derived
	*BovHHPrev*	*Leptospira* seroprevalence in bovines at household level	
	*bov_com_pos *	Number of seropositive bovines in the community	
	*bov_com_prev*	*Leptospira* seroprevalence in bovines at community level	
	*ovi_count*	Number of ovines in the household	Questionnaire
	*ovi_pos*	Presence of seropositive ovines in the household	Derived
	*OviHHPrev*	*Leptospira* seroprevalence in ovines at household level	
	*ovi_pos_com*	Number of seropositive ovines in the community	
	*OviComPrev*	*Leptospira* seroprevalence in ovines at community level	
	*dog_count*	Number of dogs in the household	Questionnaire
	*dog_pos *	Presence of seropositive dogs in the household	Derived
	*DogHHPrev*	*Leptospira* seroprevalence in dogs at household level	
	*dog_com_pos *	Number of seropositive dogs in the community	
	*DogComPrev*	*Leptospira* seroprevalence in dogs at community level	
	*Anim_pos*	Presence of seropositive animals in the household	
	*AnimalHHPrev*	*Leptospira* seroprevalence in farm animals at household level	
	*animal_pos_com*	Number of overall seropositive farm animals in the community	
	*AnimCommPrev*	*Leptospira* seroprevalence in farm animals at community level	

**Table 2:** Parameters that were used to tune the models.

Parameter	Description	Range	Interval
scale_pos_weight	Weight of positive class to address class imbalance	Neg/pos	Fixed
nrounds	Number of boosting rounds or iterations during the training process.	100–600	50
learning_rate	Learning rate for gradient boosting	0–1	0.01
max_depth	Maximum depth of the decision tree	0–10	1
min_child_weight	Minimum sum of instance weight (Hessian) needed in a child	0–10	1
gamma	Minimum loss reduction required to make a further partition on a leaf node	0–5	0.1
subsample	Fraction of training data to randomly sample during training	0–1	0.1
colsample_bytree	Fraction of features to be randomly sampled for each tree	0–1	0.1
objective	Learning task and objective function (binary classification in this case)	Binary:logistic	
Max_delta_step	Introduce an ‘absolute’ regularization, capping the weight before applying ETA correction.	1–10	0.1

## Data Availability

The data presented in this study are available on request from the corresponding author due to sensitive disease exposure, life history, and income information.

## Appendix A

**Table A1:** Seroprevalence of Leptospirosis in different demographic and behavioral factors.

Variables	Number of Participants	MAT Positive	Seroprevalence (%)	95% CI
Sex of the person				
Male	387	25	6.46	4.22–9.39
Female	520	29	5.57	3.77–7.91
Person swim				
Yes	629	43	6.84	4.99–9.09
No	278	11	3.96	1.99–6.97
Positive rodents in the household				
Yes	407	29	7.13	4.82–10.07
No	500	25	5.00	3.26–7.29
Positive dogs in the household				
Yes	128	10	7.81	4.13–7.51
No	779	44	5.64	3.81–13.90
Positive cattles in the household				
Yes	299	16	5.35	3.09–8.54
No	608	38	6.25	4.46–8.48
Positive sheep in the household				
Yes	282	13	4.61	2.48–7.75
No	625	41	6.56	4.75–8.79
Work in garden				
Yes	269	11	4.09	2.06–7.20
**No**	638	43	6.73	4.92–8.97
**Clean barn**				
**Yes**	337	23	6.82	4.37–10.06
**No**	570	31	5.44	3.73–7.63
**Clean sewage drains**				
**Yes**	46	1	2.27	0.05–11.53
**No**	861	53	6.15	4.64–7.97
**Person slaughters animals**				
**Yes**	123	9	7.31	3.40–13.43
**No**	784	45	5.74	4.22–7.60
**Person milks animals**				
**Yes**	48	4	8.33	2.32–19.98
**No**	859	50	5.82	4.35–7.60
**Clean animal at birth**				
**Yes**	96	5	5.21	1.71–11.73
**No**	811	49	6.04	4.50–7.91

## References

[1] Crecelius E.M., Burnett M.W. (2020). Leptospirosis. J. Spec. Oper. Med..

[2] Haake D.A., Levett P.N., Adler B. (2015). Leptospirosis in Humans. Leptospira and Leptospirosis. Current Topics in Microbiology and Immunology.

[3] Costa F., Hagan J.E., Calcagno J., Kane M., Torgerson P., Martinez-Silveira M.S., Stein C., Abela-Ridder B., Ko A.I. (2015). Global Morbidity and Mortality of Leptospirosis: A Systematic Review. PLoS Negl. Trop. Dis..

[4] Luna J., Salgado M., Tejeda C., Moroni M., Monti G. (2020). Assessment of Risk Factors in Synanthropic and Wild Rodents Infected by Pathogenic *Leptospira* spp. Captured in Southern Chile. Animals.

[5] Guerra M.A. (2013). Leptospirosis: Public health perspectives. Biologicals.

[6] Ellis W.A. (2015). Animal leptospirosis. Curr. Top. Microbiol. Immunol..

[7] Bradley E.A., Lockaby G. (2023). Leptospirosis and the Environment: A Review and Future Directions. Pathogens.

[8] Montes V., Monti G. (2021). Pathogenic *Leptospira* spp. Seroprevalence and Herd-Level Risk Factors Associated with Chilean Dairy Cattle. Animals.

[9] Harrison N.A., Fitzgerald W.R. (1988). Leptospirosis—Can it be a sexually transmitted disease?. Postgrad. Med. J..

[10] Bolin C.A., Koellner P. (1988). Human-to-Human Transmission of *Leptospira* interrogans by Milk. J. Infect. Dis..

[11] Muñoz-Zanzi C., Mason M.R., Encina C., Astroza A., Romero A. (2014). *Leptospira* Contamination in Household and Environmental Water in Rural Communities in Southern Chile. Int. J. Environ. Res. Public Health.

[12] Pappas G., Papadimitriou P., Siozopoulou V., Christou L., Akritidis N. (2008). The globalization of leptospirosis: Worldwide incidence trends. Int. J. Infect. Dis..

[13] Miller D.A., Wilson M.A., Beran G.W. (1991). Relationships between prevalence of *Leptospira* interrogans in cattle, and regional, climatic, and seasonal factors. Am. J. Vet. Res..

[14] Romero E.C., Bernardo C.C., Yasuda P.H. (2003). Human leptospirosis: A twenty-nine-year serological study in São Paulo, Brazil. Rev. Inst. Med. Trop. São Paulo.

[15] Mwachui M.A., Crump L., Hartskeerl R., Zinsstag J., Hattendorf J. (2015). Environmental and Behavioural Determinants of Leptospirosis Transmission: A Systematic Review. PLoS Negl. Trop. Dis..

[16] Rahman S.Z., Senthil R., Ramalingam V., Gopal R. (2023). Predicting Infectious Disease Outbreaks with Machine Learning and Epidemiological Data. J. Adv. Zool..

[17] Bi Q., E Goodman K., Kaminsky J., Lessler J. (2019). What is machine learning? A primer for the epidemiologist. Am. J. Epidemiol..

[18] Al Meslamani A.Z., Sobrino I., de la Fuente J. (2024). Machine learning in infectious diseases: Potential applications and limitations. Ann. Med..

[19] Cabrera M., Leake J., Naranjo-Torres J., Valero N., Cabrera J.C., Rodríguez-Morales A.J. (2022). Dengue Prediction in Latin America Using Machine Learning and the One Health Perspective: A Literature Review. Trop. Med. Infect. Dis..

[20] Sahu M., Gupta R., Ambasta R.K., Kumar P. (2022). Artificial intelligence and machine learning in precision medicine: A paradigm shift in big data analysis. Prog. Mol. Biol. Transl. Sci..

[21] Pandit N., Vanak A.T. (2020). Artificial Intelligence and One Health: Knowledge Bases for Causal Modeling. J. Indian Inst. Sci..

[22] Muñoz-Zanzi C., Mason M., Encina C., Gonzalez M., Berg S. (2014). Household characteristics associated with rodent presence and *Leptospira* infection in rural and urban communities from Southern Chile. Am. J. Trop. Med. Hyg..

[23] Alexander A.D. (1960). The distribution of leptospirosis in Latin America. Bull. World Health Organ..

[24] Zamora J., Riedemann S., I Montecinos M., Cabezas X. (1990). Serological survey of human leptospirosis in a high risk population in Chile. Rev. Med. Chil..

[25] Lelu M., Muñoz-Zanzi C., Higgins B., Galloway R. (2015). Seroepidemiology of leptospirosis in dogs from rural and slum communities of Los Rios Region, Chile. BMC Vet. Res..

[26] Mason M.R., Encina C., Sreevatsan S., Muñoz-Zanzi C. (2016). Distribution and Diversity of Pathogenic *Leptospira* Species in Peri-domestic Surface Waters from South Central Chile. PLoS Negl. Trop. Dis..

[27] Munoz-Zanzi C., Campbell C., Berg S. (2016). Seroepidemiology of toxoplasmosis in rural and urban communities from Los Rios Region, Chile. Infect. Ecol. Epidemiol..

[28] Fick S.E., Hijmans R.J. (2017). WorldClim 2: New 1-km spatial resolution climate surfaces for global land areas. Int. J. Climatol..

[29] Chen T., Guestrin C. XGBoost: A Scalable Tree Boosting System. Proceedings of the 22nd ACM SIGKDD International Conference on Knowledge Discovery and Data Mining 2016.

[30] Ogunleye A.A., Wang Q.-G. (2019). XGBoost Model for Chronic Kidney Disease Diagnosis. IEEE/ACM Trans. Comput. Biol. Bioinform..

[31] Shaheed K., Abbas Q., Hussain A., Qureshi I. (2023). Optimized Xception Learning Model and XgBoost Classifier for Detection of Multiclass Chest Disease from X-ray Images. Diagnostics.

[32] Ali Z.A., Abduljabbar Z.H., Taher H.A., Sallow A.B., Almufti S.M. (2023). Exploring the power of eXtreme gradient boosting algorithm in machine learning: A review. Acad. J. Nawroz Univ..

[33] Aydin Z.E., Ozturk Z.K. Performance analysis of XGboost classifier with missing data. Proceedings of the 1st International Conference on Computing and Machine Intelligence.

[34] Putatunda S., Rama K. A Comparative Analysis of Hyperopt as Against Other Approaches for Hyper-Parameter Optimization of XGBoost. Proceedings of the 2018 International Conference on Signal Processing and Machine Learning.

[35] Davagdorj K., Pham V.H., Theera-Umpon N., Ryu K.H. (2020). XGBoost-Based Framework for Smoking-Induced Noncommunicable Disease Prediction. Int. J. Environ. Res. Public Health.

[36] Srinivas P., Katarya R. (2022). hyOPTXg: OPTUNA hyper-parameter optimization framework for predicting cardiovascular disease using XGBoost. Biomed. Signal Process. Control.

[37] Farooq Z., Rocklöv J., Wallin J., Abiri N., Sewe M.O., Sjödin H., Semenza J.C. (2022). Artificial intelligence to predict West Nile virus outbreaks with eco-climatic drivers. Lancet Reg. Health Eur..

[38] Jin H., Ling C.X. (2005). Using AUC and accuracy in evaluating learning algorithms. IEEE Trans. Knowl. Data Eng..

[39] R Core Team (2020). R: A Language and Environment for Statistical Computing.

[40] Chen T., He T., Benesty M., Khotilovich V., Tang Y., Cho H., Chen K., Mitchell R., Cano I., Zhou T. (2023). XGboost: Extreme Gradient Boosting.

[41] Karpagam K.B., Ganesh B. (2020). Leptospirosis: A neglected tropical zoonotic infection of public health importance—an updated review. Eur. J. Clin. Microbiol. Infect. Dis..

[42] Notobroto H.B., Mirasa Y.A., Rahman F.S. (2021). Sociodemographic, behavioral, and environmental factors associated with the incidence of leptospirosis in highlands of Ponorogo Regency, Province of East Java, Indonesia. Clin. Epidemiol. Glob. Health.

[43] Romero M.H., A Sánchez J., Hayek L.C. (2010). Prevalencia de anticuerpos contra *Leptospira* en población urbana humana y canina del Departamento del Tolima. Rev. Salud Pública.

[44] Alvarado-Esquivel C., Hernandez-Tinoco J., Sanchez-Anguiano L.F., Ramos-Nevarez A., Cerrillo-Soto S.M., Guido-Arreola C.A. (2016). *Leptospira* Exposure and Gardeners: A Case-Control Seroprevalence Study. J. Clin. Med. Res..

[45] Benschop J., Heuer C., Jaros P., Collins-Emerson J., Midwinter A., Wilson P. (2009). Sero-prevalence of leptospirosis in workers at a New Zealand slaughterhouse. N. Z. Med. J..

[46] Carrero S.H.S., Montoya D.P.H., Bolaños Y.M., Medellín M.O.P. (2017). Seroprevalencia de infección por *Leptospira* y factores de riesgo en estudiantes de una universidad de Colombia. Nova.

[47] Dias J.P., Teixeira M.G., Costa M.C.N., Mendes C.M.C., Guimarães P., Reis M.G., Ko A., Barreto M.L. (2007). Factors associated with *Leptospira* sp infection in a large urban center in northeastern Brazil. Rev. Soc. Bras. Med. Trop..

[48] Wynwood S.J., Graham G.C., Weier S.L., Collet T.A., McKay D.B., Craig S.B. (2014). Leptospirosis from water sources. Pathog. Glob. Health.

[49] Goarant C. (2016). Leptospirosis: Risk factors and management challenges in developing countries. Res. Rep. Trop. Med..

[50] Davignon G., Cagliero J., Guentas L., Bierque E., Genthon P., Gunkel-Grillon P., Juillot F., Kainiu M., Laporte-Magoni C., Picardeau M. (2023). Leptospirosis: Toward a better understanding of the environmental lifestyle of *Leptospira*. Front. Water.

[51] Moseley M., Rahelinirina S., Rajerison M., Garin B., Piertney S., Telfer S. (2018). Mixed *Leptospira* Infections in a Diverse Reservoir Host Community, Madagascar, 2013–2015. Emerg. Infect. Dis..

[52] Cucchi K., Liu R., Collender P.A., Cheng Q., Li C., Hoover C.M., Chang H.H., Liang S., Yang C., Remais J.V. (2019). Hydroclimatic drivers of highly seasonal leptospirosis incidence suggest prominent soil reservoir of pathogenic *Leptospira* spp. in rural western China. PLoS Negl. Trop. Dis..

[53] Cunha M., Costa F., Ribeiro G.S., Carvalho M.S., Reis R.B., Nery N., Pischel L., Gouveia E.L., Santos A.C., Queiroz A. (2022). Rainfall and other meteorological factors as drivers of urban transmission of leptospirosis. PLoS Negl. Trop. Dis..

[54] Kocher A., Cornuault J., Gantier J., Manzi S., Chavy A., Girod R., Dusfour I., Forget P., Ginouves M., Prévot G. (2023). Biodiversity and vector-borne diseases: Host dilution and vector amplification occur simultaneously for Amazonian leishmaniases. Mol. Ecol..

[55] Chiani Y.T., Jacob P., Mayora G., Aquino D.S., Quintana R.D., Mesa L. (2023). Presence of *Leptospira* spp. in a Mosaic of Wetlands Used for Livestock Raising under Differing Hydroclimatic Conditions. Appl. Environ. Microbiol..

[56] Caley P., Ramsey D. (2001). Estimating disease transmission in wildlife, with emphasis on leptospirosis and bovine tuberculosis in possums, and effects of fertility control. J. Appl. Ecol..

[57] Bacallao J., Schneider M.C., Najera P., Aldighieri S., Soto A., Marquiño W., Sáenz C., Jiménez E., Moreno G., Chávez O. (2014). Socioeconomic factors and vulnerability to outbreaks of leptospirosis in Nicaragua. Int. J. Environ. Res. Public Health.

[58] Zakharova O.I., Korennoy F.I., Iashin I.V., Toropova N.N., Gogin A.E., Kolbasov D.V., Surkova G.V., Malkhazova S.M., Blokhin A.A. (2021). Ecological and Socio-Economic Determinants of Livestock Animal Leptospirosis in the Russian Arctic. Front. Vet. Sci..

[59] Baquero O.S., Machado G. (2018). Spatiotemporal dynamics and risk factors for human Leptospirosis in Brazil. Sci. Rep..

[60] Hagan J.E., Moraga P., Costa F., Capian N., Ribeiro G.S., Wunder E.A., Felzemburgh R.D.M., Reis R.B., Nery N., Santana F.S. (2016). Spatiotemporal Determinants of Urban Leptospirosis Transmission: Four-Year Prospective Cohort Study of Slum Residents in Brazil. PLoS Negl. Trop. Dis..

[61] Reis R.B., Ribeiro G.S., Felzemburgh R.D.M., Santana F.S., Mohr S., Melendez A.X.T.O., Queiroz A., Santos A.C., Ravines R.R., Tassinari W.S. (2008). Impact of environment and social gradient on *Leptospira* infection in urban slums. PLoS Negl. Trop. Dis..

[62] Kembhavi R.S., Velhal G.D., Shah A.K. (2021). Epidemiological determinants of leptospirosis in rural and urban districts of Maharashtra, India. J. Fam. Med. Prim. Care.

[63] Sluydts V., Sarathchandra S.R., Piscitelli A.P., Van Houtte N., Gryseels S., Mayer-Scholl A., Bier N.S., Htwe N.M., Jacob J. (2022). Ecology and distribution of *Leptospira* spp., reservoir hosts and environmental interaction in Sri Lanka, with identification of a new strain. PLoS Negl. Trop. Dis..

[64] Daniels M.E., Pradhan A., Odagiri M., Jenkins M.W. (2023). Waterborne exposure during non-consumptive domestic use of surface water: A population study across WASH service levels in rural India. J. Water Health.

[65] Galan D.I., Roess A.A., Pereira S.V.C., Schneider M.C. (2021). Epidemiology of human leptospirosis in urban and rural areas of Brazil, 2000–2015. PLoS ONE.

[66] Awoniyi A.M., Venegas-Vargas C., Souza F.N., Zeppelini C.G., Hacker K.P., Carvalho-Pereira T., Marins C.L., de Santana M.C., Pertile A.C., Begon M. (2022). Population dynamics of synanthropic rodents after a chemical and infrastructural intervention in an urban low-income community. Sci. Rep..

[67] Céspedes M., Ormaeche M., Condori P., Balda L., Glenny M. (2003). Prevalencia de Leptospirosis y factores de riesgo en personas con antecedentes de fiebre en la Provincia de Manu, Madre de Dios, Perú. Rev. Peru. Med. Exp. Salud Pública.

[68] Maze M.J., Cash-Goldwasser S., Rubach M.P., Biggs H.M., Galloway R.L., Sharples K.J., Allan K.J., Halliday J.E.B., Cleaveland S., Shand M.C. (2018). Risk factors for human acute leptospirosis in northern Tanzania. PLoS Negl. Trop. Dis..

[69] Brockmann S.O., Ulrich L., Piechotowski I., Wagner-Wiening C., Nöckler K., Mayer-Scholl A., Eichner M. (2016). Risk factors for human *Leptospira* seropositivity in South Germany. SpringerPlus.

[70] Anderson T., Hamond C., Haluch A., Toot K., Nally J.E., LeCount K., Schlater L.K. (2023). Animals Exposed to *Leptospira* Serogroups Not Included in Bacterins in the United States and Puerto Rico. Trop. Med. Infect. Dis..

[71] Harran E., Pinot A., Kodjo A., Djelouadji Z., Le Gudayer M., Sionfoungo Daouda S., Groud K., Lattard V., Ayral F. (2023). Identification of Pathogenic *Leptospira kirschneri* Serogroup Grippotyphosa in Water Voles (*Arvicola terrestris*) from Ruminant Pastures in Puy-de-Dôme, Central France. Pathogens.

